# Supplementary material for: Association between functional dentition and ultra-processed food consumption in Brazilian adults: A cross-sectional study
Source: PLoS One. 2025 Jun 26;20(6):e0325838. doi: 10.1371/journal.pone.0325838 (PMC12201631; doi:10.1371/journal.pone.0325838)
Supplement: Attachment — Submitted filename: Response to Reviewers.docx [file pone.0325838.s003.docx]

**Editor-in-Chief**

*Plos One*

Dear Editor-in-Chief

On behalf of all authors, I am resubmitting (R2) the revised manuscript entitled “Functional dentition and consumption of ultra-processed foods in Brazilian adults” for your consideration and editorial review at *PLOS ONE*, considering its potential for publication. We sincerely thank the editors and reviewers for the opportunity to review and improve our manuscript. All suggestions and comments provided by the reviewers and editors were carefully considered and addressed.

The authors state that this article is original and is not currently under consideration for publication in another journal. The research was approved by the National Ethics Committee and conducted under the highest ethical principles, including the well-being of human subjects and informed consent obtained from all participants. We hereby declare that we have no conflicting interests and are willing to transfer the copyright if and when the manuscript is accepted for publication.

We remain at your disposal for any further clarifications that may be necessary.

***Corresponding Author:***

Prof. Dr. Fernando Neves Hugo

Department of Epidemiology and Health Promotion, New York University, New York, NY, USA

E-mail: fnh9064@nyu.edu

Review Comments to the Author

Please use the space provided to explain your answers to the questions above. You may also include additional comments for the author, including concerns about dual publication, research ethics, or publication ethics. (Please upload your review as an attachment if it exceeds 20,000 characters)

Reviewer #1: Thank you for addressing previous comments.

Just two small ones:

**1. You have repeated the full term for PNS in abstract. Once is enough.**

**Response:** We appreciate your observation. We have revised the text as suggested, using the acronym PNS after its first full mention in the abstract.

2. Please add "Brazilian" before "adults" in the title of Table 1, as each table should be self-standing.

**Response:** Thanks for the feedback, the term has been added.

___________________________________________________________________________

Reviewer #2: (No Response)

___________________________________________________________________________

Reviewer #3: I have reviewed the revised version of the manuscript along with the authors’ responses to the editorial and reviewer comments. I appreciate the considerable effort the authors have undertaken to improve the clarity, methodological rigor, and presentation of the manuscript. The clarifications added to the methods section, improved articulation of the hypotheses, and refinement of the discussion enhance the manuscript’s scientific value and readability.

However, a few important issues remain and should be addressed before the manuscript can be considered for publication:

1. Descriptive Statistical Testing – Table 1

While the authors have now included Table 1, the p-values for comparisons across demographic and socioeconomic groups remain absent. In the initial review, I requested clarification on whether differences observed in ultra-processed food consumption across subgroups (e.g., age, sex, education) were statistically significant. Please include p-values in Table 1 to indicate whether the observed differences are statistically significant. If not included in the table, a supplementary note summarizing the key significant comparisons would suffice. This information is crucial for interpreting group-level variation and supports the argument that social determinants play a central role.

**Response:** We appreciate the suggestion to include statistical significance in Table 1. In response, we have added unadjusted prevalence ratios (PRs) for all demographic and socioeconomic comparisons in the table. All PRs with a p-value <0.05 have been highlighted in bold and explicitly mentioned in the table footnote. This adjustment ensures clarity in identifying which subgroup differences in ultra-processed food consumption are statistically significant, reinforcing the role of social determinants as discussed in the manuscript. We believe this addresses the concern while maintaining readability.

**Revised text:**

**Table 1.** Characteristics of the sample and mean of ultra-processed diet by socioeconomic factors, referring to individuals who answered about the consumption of ultra-processed diet. National Health Survey (PNS) 2019, Brazilian adults aged 18-49 years (n=50,146).

| ***Sample Characteristics*** | ***Weighted  (%)**** | ***Proportion of ultra-processed diet per exposure (CI 95%)^a^*** | ***Unadjusted***  ***PR^a^ (95% CI)**** |
| --- | --- | --- | --- |
| ***Socioeconomic factors*** |  |  |  |
| Zone |  |  |  |
| Urban | 13.8 | 19.4 (18.7 - 20.2) | **1** |
| Rural | 86.2 | 9.9 (9.0 – 10.9) | **1.08 (1.07 – 1.09)^b^** |
| Sex |  |  |  |
| Male | 47.8 | 19.7 (18.7 – 20.7) | **1** |
| Female | 52.1 | 16.6 (15.8 – 17.5) | **0.97 (0.96 – 0.98)^b^** |
| Skin Color |  |  |  |
| White | 39.8 | 19.4 (18.3 – 20.5) | **1** |
| No White | 60.1 | 17.3 (16.5 – 18.0) | **0.98 (0.97 – 0.99)^b^** |
| Age (years) |  |  |  |
| 18-29 | 36.7 | 23.0 (21.7 – 24.3) | **1** |
| 30-39 | 34.2 | 17.7 (16.7 – 18.7) | **0.95 (0.94 – 0.97)^b^** |
| 40-49 | 29.6 | 12.7 (11.8 – 13.8) | **0.91 (0.90 – 0.92)^b^** |
| Formal education |  |  |  |
| ≤ 8 Years | 29.4 | 14.3 (13.3 – 15.3) | **1** |
| > 8 Years | 70.5 | 19.7 (18.9 – 20.5) | **1.04 (1.03 – 1.05)^b^** |
| Household income ^a^ |  |  |  |
| Up to 1 Minimum wage (MW) | 56.1 | 15.7 (14.4 – 17.0) | **1** |
| More than 1 up to 3 (MW) | 34.1 | 20.5 (18.7 – 22.4) | **1.02 (1.01 – 1.04)^b^** |
| More than 3 up to 5 (MW) | 5.4 | 18.3 (15.7 – 21.2) | 1.01 (0.98 – 1.03) |
| More than 5 (MW) | 4.1 | 13.1 (10.7 – 16.0) | **0.96 (0.94 – 0.99)^b^** |
| Use of dental service in the last 12 moth |  |  |  |
| No use | 45.3 | 17.4 (16.5 – 18.4) | **1** |
| Use | 54.6 | 18.7 (17.8 – 19.6) | **1.01 (1.00 – 1.02)^b^** |
| Funtional dentition |  |  |  |
| 19 or less teeth | 1.5 | 13.8 (7.4 – 24.2) | 1 |
| 20 or more teeth | 98.4 | 18.2 (17.5 – 18.8) | 0.96 (0.89 – 1.03) |

*Taking into account the sample weight.

^a^95% CI, 95% confidence interval.

^b^ *p*-value < 0.05

2. Figure 1 Caption and Terminology

The title of Figure 1 still reads as a sentence ("Representation of the Directed Acyclic Diagram (DAG)...") rather than a concise, telegraphic label. Please revise the figure caption to reflect standard scientific format. A recommended revision might be:

"Figure 1. Directed acyclic graph (DAG) illustrating hypothesized confounders in the relationship between functional dentition and ultra-processed food consumption."

**Response:** Thanks for the suggestion, the title has been changed as suggested to "Figure 1. Directed acyclic graph (DAG) illustrating hypothetical confounders in the relationship between functional dentition and ultra-processed food consumption."

3. Additional Discussion of Socioeconomic Findings (Optional but Recommended)

Although the authors explain that covariates were included as confounders, it would strengthen the discussion to briefly summarize which socioeconomic factors (e.g., education, income) were most strongly associated with ultra-processed food consumption in the adjusted model—even if these were not the primary variables of interest. Consider briefly commenting on the adjusted prevalence ratios for key covariates to contextualize the social gradient in food consumption and reinforce the policy implications.

**Response: We appreciate the suggestion. As recommended, we expanded the discussion to include a brief description of the main findings related to socioeconomic variables, even though these are not the main focus of the study. In the revised text, we highlight that lower levels of education and income were classified as positively associated with the consumption of ultra-processed foods, even after adjusting for functional dentition and other covariates. This inclusion contributes to strengthening the existence of a social gradient in eating behavior and its implications for public health. In addition, we have included a new Appendix I, which presents the complete results of the adjusted analysis, including prevalence ratios and respective confidence differences for all variables included in the model. This table allows a detailed visualization of the effects of the covariates considering the multivariate regression.**

**Revised text:**

**3rd paragraph of the discussion**

*It is important to consider that socioeconomic and behavioral factors may play a more significant role in food choice than the number of teeth.^20^ The inclusion of covariates such as income, education, urban/rural setting, and use of dental services in the analysis may have diluted the association between functional dentition and diet, as these factors can act as direct determinants in the choice of ultra-processed foods.^7,23^ The results also revealed that* ***lower levels of education and income were consistently associated with higher consumption of ultra-processed foods****, even after adjusting for dental status and other covariates, reflecting a clear social gradient.* ***This finding reinforces the notion that structural determinants of health strongly influence dietary behavior****, independent of oral health status (Appendix I). Eating behavior, shaped by cultural norms and economic constraints, may mediate this relationship, suggesting that a more comprehensive exploration of these pathways would be valuable in future research.^24 – 26^*

Appendix I. Adjusted association between ultra-processed diet and functional dentition in Brazilian adults aged 18-49 years (n=50,146) through Poisson regression with robust variance.

| ***Sample Characteristics*** | ***Adjusted***  ***PR^a^ (95% CI)**** |
| --- | --- |
| ***Socioeconomic factors*** |  |
| Zone |  |
| Urban | **1** |
| Rural | **1.07 (1.06 – 1.08)^b^** |
| Sex |  |
| Male | **1** |
| Female | **0.96 (0.95 – 0.97)^b^** |
| Skin Color |  |
| White | **1** |
| No White | **0.98 (0.97 – 0.99)^b^** |
| Age (years) |  |
| 18-29 | **1** |
| 30-39 | **0.95 (0.94 – 0.97)^b^** |
| 40-49 | **0.92 (0.90 – 0.93)^b^** |
| Formal education |  |
| ≤ 8 Years | **1** |
| > 8 Years | **1.01 (1.00 – 1.03)^b^** |
| Household income ^a^ |  |
| Up to 1 Minimum wage (MW) | **1** |
| More than 1 up to 3 (MW) | 1.01 (0.99 – 1.02) |
| More than 3 up to 5 (MW) | 0.98 (0.96 – 1.01) |
| More than 5 (MW) | **0.94 (0.92 – 0.96)^b^** |
| Use of dental service in the last 12 moth |  |
| No use | **1** |
| Use | 1.00 (0.99 – 1.02) |
| Funtional dentition |  |
| 19 or less teeth | 1 |
| 20 or more teeth | 1.02 (0.94 – 1.09) |

^a^PR, prevalence ratio; ^b^95% CI, 95% confidence interval.

*Taking into account the sample weight.

^b^ *p*-value < 0.05

4. Minor Language Polishing

The authors have addressed many language concerns, and the manuscript reads more clearly. However, I recommend a final round of professional English editing to ensure fluency, especially in the Abstract and Discussion sections.

**Response:** We thank the reviewer for the careful reading. In response to the recommendation, the manuscript has undergone a final language revision by a professional English. We have carefully revised the text, with particular attention to the Abstract and Discussion sections, to improve clarity, coherence, and overall fluency.
